# MSCs: Delivery Routes and Engraftment, Cell-Targeting Strategies, and Immune Modulation

**DOI:** 10.1155/2013/732742

**Published:** 2013-08-13

**Authors:** Thomas J. Kean, Paul Lin, Arnold I. Caplan, James E. Dennis

**Affiliations:** ^1^Benaroya Research Institute, Seattle, WA 98101, USA; ^2^Skeletal Research Center, Department of Biology, Case Western Reserve University, Cleveland, OH 44106, USA

## Abstract

Mesenchymal stem cells (MSCs) are currently being widely investigated both in the lab and in clinical trials for multiple disease states. The differentiation, trophic, and immunomodulatory characteristics of MSCs contribute to their therapeutic effects. Another often overlooked factor related to efficacy is the degree of engraftment. When reported, engraftment is generally low and transient in nature. MSC delivery methods should be tailored to the lesion being treated, which may be local or systemic, and customized to the mechanism of action of the MSCs, which can also be local or systemic. Engraftment efficiency is enhanced by using intra-arterial delivery instead of intravenous delivery, thus avoiding the “first-pass” accumulation of MSCs in the lung. Several methodologies to target MSCs to specific organs are being developed. These cell targeting methodologies focus on the modification of cell surface molecules through chemical, genetic, and coating techniques to promote selective adherence to particular organs or tissues. Future improvements in targeting and delivery methodologies to improve engraftment are expected to improve therapeutic results, extend the duration of efficacy, and reduce the effective (MSC) therapeutic dose.

## 1. Introduction

Mesenchymal stem cells (MSCs) are multipotential adult progenitor cells that have the capacity to differentiate along several mesenchymal lineages, including cartilage, adipose, marrow stroma, and bone tissue [1–3]. Studies have been conducted on the use of MSCs as a therapeutic based on this capacity to differentiate directly into these end-stage phenotypes, including the use of MSCs to promote or augment bone repair [4] and for the repair of cartilage defects [4, 5]. In addition to direct differentiation into end-stage phenotypes, MSCs have also been shown to have a positive therapeutic effect in many repair situations because of their capacity to secrete trophic factors (reviewed in [6]) that contribute to repair via the promotion of vascularization and the inhibition of cell death as well as through the modulation of the immune response. Currently, there are over 160 open studies and 116 closed clinical trials (results retrieved (3rd June 2013) in a search of www.clinicaltrials.gov on the search term “mesenchymal stem cells” and excluding trials with an unknown status and those that were conducted *in vitro*) that use MSCs to treat a variety of conditions that range from direct formation of bone tissue to treatments for graft versus host disease (GvHD) [7–9], myocardial infarction, brain trauma, and multiple sclerosis [10, 11] (reviewed by Millard and Fisk [12]). Indeed, MSCs have been well characterized with respect to their ability to produce a range of growth factors and cytokines, which inspired the designation of these cells as a kind of *“injury drugstore”* [13]. An interesting subset of this factory of cytokines is the factors that have been shown to have a profound effect on modulating the immune system. These immune modulatory factors are being tested for their effect on immune disorders such as GvHD, rheumatoid arthritis [14, 15], multiple sclerosis [16, 17], type I diabetes [18, 19], inflammatory bowel disease (IBD) [20–23], and transplant tolerance [24]. 

Of particular relevance to the therapeutic application of MSCs is their fate post-implantation. Ambiguity seen in the efficacy of MSCs, in both animal studies and clinical trials, with therapies being ineffective or only temporarily effective could be due to suboptimal application of MSCs. Whether systemically injected or injected directly into a tissue or organ, there is the issue of where the cells go and whether the cells can bind, engraft, and, in many instances, survive. Very few studies have quantified the efficiency of MSC transplantation, and those that have quantified MSC engraftment have shown poor engraftment efficiency. Complicating this determination, as noted in Karp and Leng Teo [25], are the details of the quantification methodology. The techniques for assessing biodistribution of MSCs can be categorized into *in vivo* and *ex vivo *methods. Examples of *in vivo* methods include bioluminescence, whereby cells are transduced to express luciferase and can then be imaged through their metabolism of luciferin resulting in light emission [26]; fluorescence, whereby cells are either loaded with a fluorescent dye or transduced to express a fluorescent reporter which can then be imaged; radionuclide labeling, where cells are loaded with radionuclides and localized with scintigraphy [27], positron emission tomography (PET) or single photon emission computed tomography (SPECT); and magnetic resonance imaging (MRI), wherein cells loaded with paramagnetic compounds (e.g., iron oxide nanoparticles) are traced with an MRI scanner. For further review of these imaging modalities and their clinical application see Srinivas et al. [28] and Reagan and Kaplan [29]. *Ex vivo* methods to assess biodistribution include quantitative PCR, flow cytometry, and histological methods. Histological methods include tracking fluorescently labeled cells; *in situ* hybridization, for example, for Y-chromosomes and for human-specific ALU sequences; and histochemical staining for species-specific or genetically introduced proteins such as bacterial *β*-galactosidase. These immunohistochemical methods are useful for discerning engraftment location but necessitate the excision of tissue. They are, however, prone to errors for quantification due to sampling, the possibility of false positives, and the loss of signal in studies where MSCs are tagged with fluorescent probes that lose signal with each cell division. With the use of genetic markers, such as luciferase or green fluorescent protein (GFP), the fate of MSCs in whole animals can be tracked without losing signal after cell division. However, luciferase and GFP-like optical probes suffer from limited penetration and 3D localization issues due to tissue shielding; thus, they are most applicable in small animal models, while combinational tracking approaches make it feasible to more accurately track cell fate systemically and over extended periods of time. Additionally, care must be taken in how the cells are labeled, as certain methods of labeling MSCs with reporter genes can affect their proliferation [26] or their differentiation potential [30]. With the use of these newer tracking methods, researchers now have the ability to address the long-term fate of transplanted MSCs, although there is still a paucity of data related to this issue. This review will analyze factors that may influence the therapeutic efficacy of MSCs, including an overview of the immune status of MSCs, “intrinsic” MSC activity, optimal MSC delivery methods, and targeting methods to improve cell engraftment and survival. 

## 2. MSC Immune Modulation

Several characteristics of MSCs are purported to impart immune privilege, thereby allowing MSCs to avoid immune rejection in certain situations, which may facilitate the clinical use of allogenic MSCs. MSCs do not express class II Major Histocompatibility Complex (MHC) or costimulator molecules and express low levels of class I MHC [31]. One of the first indicators of the role of MSCs in modulating immune reactions was in studies showing that activated MSCs inhibit T-cell expansion in mixed lymphocyte reactions [32–34]. MSCs have been shown to influence the immune system through the secretion of a variety of soluble factors including indoleamine 2,3-dioxygenase [35], nitric oxide [9], transforming growth factor beta (TGF*β*), prostaglandin E2 (PGE2) [36, 37], and tumor necrosis factor stimulated gene-6 protein (TSG-6) [38]. Di Nicola et al. [39] showed that MSCs could impede T-cell expansion in transwell cultures and that this inhibitory effect was abolished by the addition of antibodies to TGF*β* and hepatocyte growth factor (HGF), while Tse et al. [33] showed a similar inhibitory effect that was a soluble factor but not due to TGF*β*, PGE2, or interleukin-10. In still another study, this time using adipose-derived MSCs, it was shown that the inhibition of T-cell expansion in mixed lymphocyte reactions was abrogated by the addition of indomethacin, a PGE2 inhibitor, but was not affected by antibodies to TGF*β* or HGF [40]. It has also been demonstrated that proinflammatory factors, such as interferon-*γ* or tumor necrosis factor alpha (TNF*α*), upregulate the expression of these important regulatory factors [9, 35, 41]. This MSC anti-inflammatory response to proinflammatory stimulation is referred to as “licensing” [35]. For a detailed review on the mechanism of action of each of these factors, see English (2013) [42]. 

While many studies have shown that soluble factors play a significant role in the immunomodulatory characteristics of MSCs, several other studies indicate that cell-to-cell contact may also be important. In one study, MSCs were shown to inhibit proliferation of memory T-cells but, in this case, direct cell contact was required as the MSCs were ineffective in transwell experiments [35]. Another study showed that MSCs interact with T-cells via Notch signaling and that inactivation of Toll-3 and Toll-4 receptors downregulated Jagged-1 expression in MSCs, thus impeding interaction with Notch on T-cells and resulting in the loss of MSC inhibition of T-cell proliferation [43]. Indeed, several more recent studies indicate that cell-to-cell contact between MSCs and immune cells may be very important in specific situations. For example, it was shown that tissue-resident MSCs are able to promote epithelial cell repair through the secretion of PGE2 after the MSCs had been exposed to microbes of the GI-tract, while mice deficient in Toll-like receptor signaling (myeloid differentiation primary response gene 88 knockouts; Myd88^−/−^) were ineffective at epithelial repair [44]. Additional studies have shown that MSCs may also regulate dendritic cells via Notch signaling [45, 46]. 

The mechanism of the immunomodulation characteristics of MSCs is critical when considering the potential therapeutic effects of MSC transplantation. If MSCs need cell-to-cell contact or need to secrete factors at a high local concentration to impart their regulatory role, then MSCs will need to be delivered in close proximity to the target tissue or organ. Alternatively, MSCs may act distally through the secretion of sufficient amounts of soluble factors with an adequate half-life to reach the target lesion. For example, TSG-6 was shown to play a key role in MSC-mediated inflammatory regulation in postmyocardial infarction. In this study, MSCs or TSG-6 alone, but not MSCs treated with TSG-6 siRNA, were able to decrease postmyocardial infarction inflammation and improve cardiac function [38]. A similar anti-inflammatory effect of MSC-produced TSG-6 was shown in a cornea transplant model [47], and another study in a peritonitis model showed that MSCs stimulated by macrophage-produced TNF*α* produced TSG-6 which acted as a negative feedback loop on macrophage inflammatory signaling [48]. 

These immunomodulatory characteristics are likely the underlying mechanism(s) of the anti-inflammatory role MSCs play in many of the aforementioned clinical trials. However, this purported ability to avoid rejection remains controversial, with some studies showing rejection in allogenic settings and others showing tolerance, and a selection showing rejection when MSCs begin to differentiate (see review by Griffin et al. [49]). In one study, it was shown that when allogeneic rat MSCs were combined with ceramics and implanted subcutaneously to promote osteoblast differentiation, the MSCs elicited an immune response and were rejected [50], but the MSCs were able to form bone in the ceramic if the host rats were administered an immunosuppressive drug (FK-506). In a rat cardiac study, it was also shown that transplanted MSCs expressing a cardiac phenotype were responsible for the induction of a host immune response and subsequent rejection [51]. In another study, it was also shown that MSCs are unable to avoid detection by the innate immune system and that cell culture conditions alone may impart a change in MSCs that allows innate recognition of autotransplanted MSCs [52]. This rejection issue is an important aspect of developing effective therapies because if most or all of the MSCs are rejected, then the treatment may not be as effective or long lasting. However, if allogeneic MSCs are well tolerated, it would be easier and less expensive to use banked allogeneic MSCs than it would be to use autologous MSCs. If MSCs are rejected after they express class II MHC markers, it does not preclude the use of allogeneic MSCs in cases where MSCs are not expected to differentiate. In fact, many studies have shown that allogeneic MSCs are well tolerated in people, and many of the positive results observed in various clinical trials have come from patients transplanted with allogeneic MSCs (reviewed by Millard and Fisk [12]). However, it may be that, in many studies, the observed therapeutic effects do not require long-term engraftment. In a study examining the long-term engraftment of allotransplanted MSCs in deceased patients having received MSC infusions, it was shown that only small numbers, if any, of the infused MSCs were detectable [53]. This observation has led to the proposal that some MSC therapeutic effects are essentially “hit and run” phenomena. The fact that MSCs often do not engraft seems a likely explanation for why many MSC therapies are marginally or only transiently effective. Indeed, it could be speculated that the initial improvement in cardiac function seen at a 6-month time point in the BOOST clinical trial, that had disappeared by the 18-month time point, could have been extended with improved engraftment [54]. Clinically, if MSCs were to be used in conditions where they might need to differentiate in order to achieve a therapeutic effect or need to be engrafted, the use of autologous MSCs is a preferred option. Whether MSCs are immunomodulatory or used as an autograft, there is the critical issue of effectively delivering them to the site of action and, ideally, enabling engraftment.

## 3. MSCs, Pericytes, and Tissue Repair

At sites of injury, it has been established that most, if not all, MSCs are derived from perivascular mesenchymal cells, pericytes, that are on the tissue side of blood vessels and sinusoids [55, 56]. This location has been identified in MSCs derived from bone marrow and dental pulp [57], brain [58], umbilical cord [59], adipose tissue [60, 61], and liver [62]. Whether all pericytes are MSCs or whether MSCs are the bone marrow-derived subset of pericytes is debatable since “pericytes” or “MSCs” isolated from different organs of the body can display markedly different differentiation potentials, such as pulp-derived pericytes/MSCs displaying odontoblastic potential [63], while marrow-derived pericyte/MSCs have not shown an odontoblastic phenotype. The argument is that if all pericytes were MSCs, then all pericytes should have equivalent differentiation potentials. Alternatively, it has been proposed that pericytes constitute a reservoir of tissue-specific progenitor cells [64], of which classically defined MSCs may only be a subset. 

Regardless of their designation as either pericytes or MSCs, it has been proposed that, during injury, blood vessels break or become inflamed and the pericytes are liberated from their perivascular tethers [65]. These released pericytes become activated and function to modulate the local immune environment and establish a zone of tissue regeneration. It is apparent, in some cases, that this release is a local effect and there is little cell recruitment from uninjured tissue [66]. There are, however, a number of publications that provide evidence that MSCs home to sites of inflammation or tissue injury when infused into living organisms. Thus, it can be inferred that in some injured tissues, MSCs may have an intrinsic affinity for the sites of tissue injury. Several studies have shown that MSCs respond to chemokines, such as SDF-1 [67], MCP-3 [68], CXCL9, CXCL16, CCL20, CCL25 [69], and HGF [70], which can either act locally or recruit MSCs from the bloodstream (the issue of systemic MSC mobilization will not be discussed further here).

The injected or naturally released MSCs are activated by the local microenvironment and respond to these cues by secreting a site-specific array of bioactive molecules. These molecules act to immunomodulate the MSC microenvironment, reduce inflammation, and establish a regenerative milieu. We would further assert that these MSCs are geared to function in the repair of these compromised vessels. Indeed, MSCs are capable not only of stimulating angiogenesis in injured muscle by secreting large amounts of vascular endothelial growth factor that attracts vascular progenitors and is cytoprotective [71], but also of stabilizing newly formed capillaries [72] by assuming perivascular positions embedded in the basement membranes of newly forming blood vessels.

## 4. MSC Delivery and Biodistribution

There are two principal methods to introduce cells into the body: local delivery into the tissue and systemic delivery. Local delivery can be further defined by the specific type of delivery, either cells embedded in a scaffold (for review see Arthur et al. [73]) or local injection, for example intraperitoneal (IP), intramuscular, or intracardiac injection. Systemic delivery can be further defined by the vascular route, venous (IV) or arterial (IA). The optimal method of delivery will depend on which mechanism of action of the MSC is being utilized. This review will concentrate on the distribution of injected cells.

Having defined some of the characteristics and functions of MSCs, the question then becomes whether or not MSCs perform best when present at the site of injury/inflammation. If MSCs can exert their effect distally, for example, by secreting cytokines into the circulation [6, 13, 38], then it may not be necessary for the cells to be located at the specific injury site and systemic effects could be achieved using a cell reservoir. For example, when MSCs were injected IP, they were able to prevent the damage caused by collagen-induced arthritis, despite the lack of a detectable presence in the arthritic joints [74]. If, however, MSCs need to be present at the site of injury, for example, by differentiating into replacement cells, as in the treatment of osteogenesis imperfecta (OI) [75] or by the local production of antiapoptotic or angiogenic factors [6], then the delivery system must place cells at, or allow MSCs to migrate to, the site of injury.

Arguments for local injection include not only delivering MSCs to the site of the lesion, but also the possibility that the MSCs have the capacity to migrate toward injured tissue. For example, when MSCs were injected IP, they were found to migrate toward an inflamed colon [76]. While IP injection is a common systemic delivery route for small molecules, IP injected MSCs [76] and neuroprogenitor cells [77] were not found to migrate to other tissues. However, it is possible that lymphoid access could be achieved through this route, leading to widespread distribution of a subset of the injected cells detected by endpoint PCR [78]. In another example, MSCs were found to migrate toward an ischemic lesion of the brain when injected near the location of the ischemia and appeared, morphologically, to differentiate into microglia [79]. MSCs have also been found to migrate toward tumors *in vivo*; it is hypothesized that tumors mimic a chronic wound [80]. When MSCs were directly injected into the brain, they were found to migrate to glioma cells located on the contralateral hemisphere [81]. The disadvantage of local injection is that it can lead to further tissue damage from the injection bolus [69]. It has also been shown with bone marrow mononuclear cells that, although direct injection increased localization, it did not increase engraftment or survival [82]. Furthermore, there has been little evidence that MSCs can migrate out of local tissues and into the circulatory system, which is problematic if MSCs need to be present in multiple body compartments, or if the injury is systemic.

**Figure 1:**
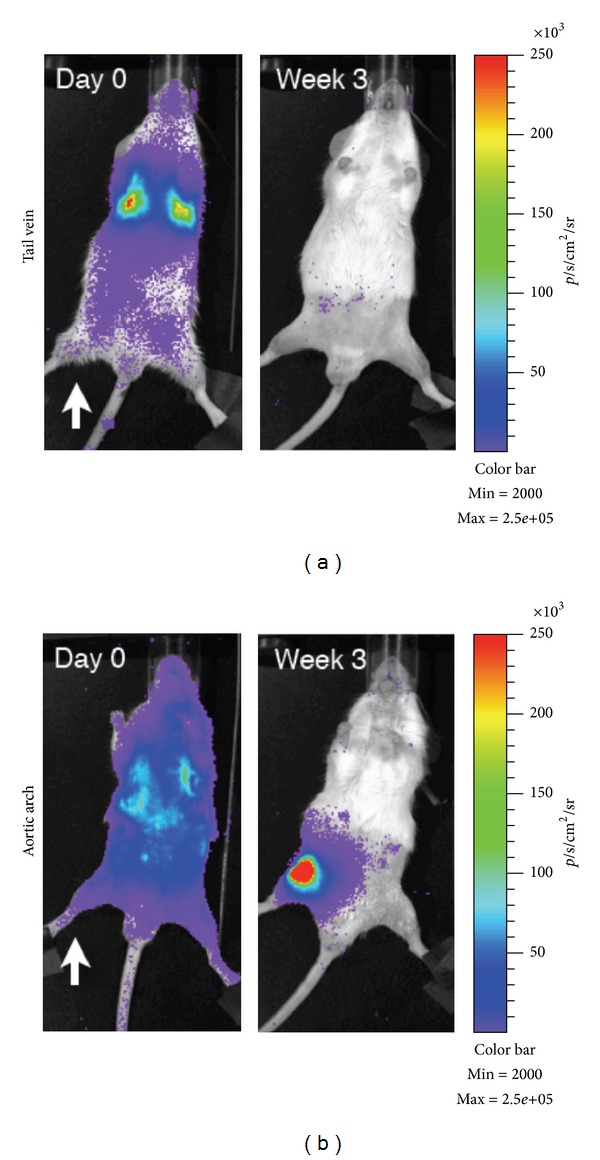
Comparison of intravenous and intra-arterial cell delivery. Mice were irradiated unilaterally (white arrow) before injection IV (tail vein; a) or IA (aortic arch; b) with 1 × 10^6^ BMC9 MSCs expressing a luciferase reporter.

The alternative method is to deliver the cells intravascularly. With this systemic injection methodology, there remain several hurdles to overcome in order to deliver cells to the target tissue and have them engraft. Intravascular injection has the advantage of being minimally invasive, and it allows for wide distribution of cells throughout the body. The most common method for accessing the circulatory system is IV and is historically the most common method for delivering MSCs. However, cells delivered IV have to first pass through the lungs before they can distribute throughout the body. This presents a major problem with what has been termed the pulmonary “first-pass” effect, which results in significant entrapment of cells [83]. This problem arises because MSCs have an estimated diameter of 20–30 *μ*m [27, 83, 84] and experiments with microspheres have demonstrated that most particles of this size are filtered out by the lungs [83, 85]. While this may be an overestimation of the amount of entrapment, as microspheres are rigid and MSCs can deform, experimental data supports that a large proportion of MSCs are entrapped in the lung following IV administration [27] (Figure 1). Furthermore, it was observed that the number of trapped cells decreased with the administration of a vasodilator [27], lending support to the hypothesis that MSC size is a major contributor to the first-pass effect. In addition to size, it is possible that endothelial cell adhesion molecules contribute to lung entrapment. This hypothesis is supported by evidence showing that when the CD49d receptor was blocked, there was a small, but significant, decrease in the number of cells trapped in the lungs [83]. In a comparison between umbilical cord blood-derived MSCs (UCB-MSCs) and bone marrow-derived MSCs (BM-MSCs), a significant difference was found in the cell surface expression of adhesion molecules (significantly higher CD49f, CD49d, and cMET in UCB-MSCs) and glycolipid carbohydrate epitopes (significantly lower GD2 and SSEA4 in UCB-MSCs), and this cell surface profile correlated with lung clearance rates, with UCB-MSCs exiting the lungs faster than BM-MSCs [86]. 

While the previous examples were from animal models, it is likely that lung entrapment is also a hindrance to MSCs administered to humans. For example, when MSCs were administered IV into OI patients, less than 1% of the cells were detected in the target organ by PCR [87, 88]. Similarly low MSC engraftment levels were found with IV administration of MSCs to treat GvHD [53], or when co-infused with hematopoietic stem cells (HSCs) to promote HSC engraftment [89]. However, it is difficult to ascertain from these two studies whether lung entrapment, or some other mechanism such as cell death, was the cause of the low engraftment efficiency since the injected cells were not tracked in real time. 

There has been some suggestion that MSC entrapment in the lungs itself can potentially be therapeutic. In experiments by Lee et al. [38], MSCs trapped in the lungs were still able to improve cardiac function after a myocardial infarction through the release of the anti-inflammatory protein TSG-6. However, experiments with OI patients were not 100% successful [87], and experiments with IV-delivered MSCs in a mouse model require the use of at least 1 × 10^6^ cells and, more frequently, a dose as high as 5 × 10^6^ cells/mouse to observe any effect. This suggests that in certain circumstances, higher absolute numbers of cells are needed to ensure that a minimum number of cells reach the injury site distal to the lungs. This cell dosing issue has important clinical significance [90], as the vast majority of human clinical trials have been for safety, with efficacy as a secondary endpoint. Little efficacy data has been shown [91] and none reported as yet on clinicaltrials.gov. Across all indications, there is a broad range in the number of cells administered. Of the 276 clinical trials mentioned in our introduction, 59 give a dose per injection per person with a mean of 2.16 × 10^8^ cells/person (1 × 10^6^, 5 × 10^7^, 5 × 10^9^; minimum, median, and maximum, resp.) and 75 give a dose/kg with a mean of 7.24 × 10^6^ cells/kg (1 × 10^5^, 2 × 10^6^, 2 × 10^8^; minimum, median, and maximum, resp.). For instance, Horwitz et al. used a cell dose of 1–5 × 10^6^/kg in treating OI pediatric patients [87], and Le Blanc et al. used a cell dose of 1-2 × 10^6^/kg in treating patients with GvHD [92]. Similar to pharmacological studies, in which there is an effective dose (ED) for drug treatment, there is an effective cell dose (ECD) equivalent for cell therapy, which is defined as the minimum cell dose required to discern a significant therapeutic effect. To put this in perspective, a commonly used cell dose of 1 × 10^6^/30 g mouse would be equivalent to 33 × 10^6^/kg or approximately 2.3 billion cells for a 70 kg human. This number is technically and operationally challenging, and most MSC therapies, as part of ongoing clinical trials in humans, use significantly lower cell doses. 

Arterial delivery has the potential to alleviate some of these dosing limitations. In theory, arterial delivery allows the cells to bypass the lungs at least once and thus avoid the pulmonary first-pass effect. This venous versus arterial effect has long been recognized in other model systems. For example, to obtain bone engraftment of melanoma cells, tumor cells were injected into the arterial system [93]. If delivered IV, tumor engraftment was primarily seen only in the lungs. Other groups have attempted a similar tactic to deliver MSCs with similar results. For example, Tögel et al. [94, 95] found increased numbers of IA-infused MSCs in kidneys in an acute kidney injury model after 24 h. This was also true in another study: when MSCs were infused IA in a model of acute stroke, it resulted in enhanced cerebral MSC engraftment [96]. 

Our experiments with delivering MSCs into the arterial system by injection into the aortic arch or tail vein support this “first-pass” cell delivery hypothesis. In this study designed to test for homing to injured tissue, mice were injured prior to injection by irradiating only one leg and were then injected with MSCs transduced with a luciferase reporter gene and the signal tracked over time. As expected, MSCs showed significant entrapment in the lungs when delivered IV into the tail vein. However, when delivered IA through the aortic arch, the cells were more evenly distributed throughout the entire animal (Figure 1). Over time, there was evidence of engraftment of the MSCs at the injury site and not at the contralateral, noninjured leg in the arterial-delivered groups. In the tail vein-injected group, the cells dissipated from the animal after a few days and were undetectable one week after injection. 

In a separate set of studies, we were able to decrease the ECD to 2.5 × 10^5^ cells/200 *μ*L/mouse (fourfold ≤ original dose), by simply switching from IV to IA delivery (unpublished results). This has important consequences. First, there is concern with cell therapies of creating a cell embolus, even with IA delivery, that could potentially lead to increased mortality [96–98]. These concerns depend on both the concentration and rate of cell delivery [97]. We, and others [99], have found pulmonary emboli and increased mortality in IV-delivered animals at higher cell dose/flow rate. In particular, concentrations above 1.0 × 10^7^ cells/mL by IV administration in mice lead to insufficient dilution of the cells in the blood, resulting in occlusion in the first capillary bed that is encountered (i.e., pulmonary embolism) and a significant increase in mortality. IA delivery is capable of decreasing the dose necessary, administers into a higher flow rate causing greater dilution, and thus decreases the potential risk of cellular embolism. Furthermore, for clinical applications, this reduction in cell dosage translates into a cell dose of ~8 × 10^6^/kg for an average human which is much more feasible than the 33 × 10^6^/kg ECD calculated previously. It should be noted that, while this dose is within the realm of possibility for pediatric patients, this still equates to ~560 × 10^6^ MSCs/70 kg adult, which remains a challenge clinically. As a consequence, further improvements to MSC engraftment are needed.

## 5. Cell Targeting Strategies 

Although there are numerous reports of stem cells homing to injured tissue, the mechanisms have yet to be elucidated and could be heavily reliant on the leaky vasculature found in injured [100, 101] or tumor tissue [102] resulting in passive entrapment in the interstitial space. It is also evident that any endogenous homing is insufficient, with less than 1% of delivered cells being found in target tissues [103]. With this in mind, we now review various methods that have been employed to enhance site-specific delivery using cell surface ligands.

### 5.1. Targeting Approaches

As with the application of ligand-directed techniques to drug therapies [104], there are a variety of mechanisms which are being investigated to enhance endogenous cell homing. In contrast to synthetic molecules, the cell represents a much more complex and dynamic environment, conferring both advantages and disadvantages. On the positive side, cells are a self-contained manufacturing plant capable of synthesizing therapeutic molecules, sensing the environment, and responding to signals orchestrating regeneration or repair. On the negative side, cells are a difficult product to characterize; more expensive to produce; have nonspecific or unwanted cell-cell interactions; and can internalize targeting ligands applied to the cell surface. The targeting approaches described in the following section are categorized as antibody-, genetically-, selectin-, and peptide-directed cell therapies. For further review of cell surface modification strategies see Stephan and Irvine [105].

### 5.2. Antibody-Directed Cell Therapy

Antibodies are highly specific ligands with excellent affinity for their antigen. Antibodies have, therefore, found application in producing the first approved ligand-targeted therapeutic, gemtuzumab ozogamicin, an anti-CD33 monoclonal drug conjugate for the treatment of myeloid leukemia [106, 107]. It should be noted that the FDA withdrew approval due to safety concerns in 2010, but clinical trials continue, and the European Medicines Agency has given it an orphan designation (EU/3/00/005). Other antibody-drug conjugates have progressed through phase III clinical trials with positive results, for example, trastuzumab emtansine, an anti-human epidermal growth factor receptor 2-targeted taxane to treat breast cancer [108], which has recently gained FDA approval (Feb. 2013). This same antibody targeting rationale has been applied to the delivery of stem cells. Two methods of antibody attachment to the cell surface have been investigated: the use of lipidated protein-G followed by antibody incubation [109–111] and bispecific antibodies [112–114].

**Figure 2:**
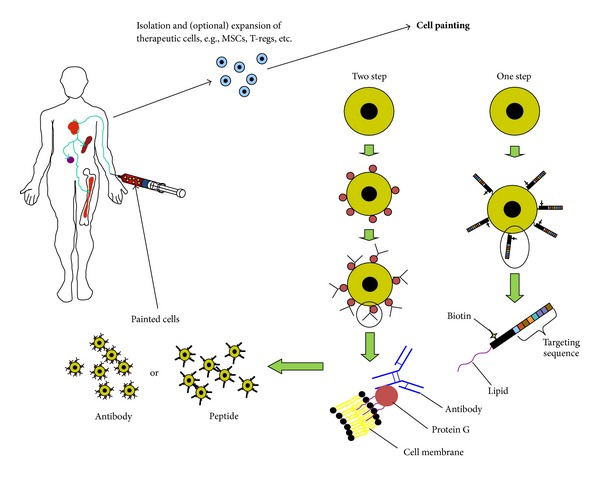
Antibody- and peptide-based cell painting. Therapeutic cells are harvested and “painted” either by a two-step process where the cells are first coated with lipidated protein A (or G) and then incubated with targeting antibody, or a one-step process where the cells are coated with a peptide-targeting molecule that contains a lipid moiety. The coated cells are then reintroduced into the patient where the cells are directed towards specific tissues or organs.

Lipidation has been investigated using palmitated protein A [115] and palmitated protein-G (PPG) [109–111], both of which bind to the Fc domain of antibodies with high affinity, but with differing selectivity based on the immunoglobulin subtypes and species in which the immunoglobulin was raised. This process has been termed “cell painting” [116] and is outlined in Figure 2 [111]. In these studies, we achieved increased cartilage progenitor cell numbers in a cartilage defect with PPG antibody-painted cells over unpainted controls [111]. In more recent work, we have shown that anti-intercellular adhesion molecule-1 (ICAM-1) and anti-vascular cell adhesion molecule-1 (VCAM-1) antibodies can be used to increase cell adherence in antigen-coated plates and in human vascular endothelial cell monolayers stimulated to express ICAM-1 with TNF*α*. This increase in attached cells is even more apparent when cells are subjected to shear at physiological levels [109]. Cells, in these examples, MSCs, are first coated with PPG by incubating with a physiological buffer containing PPG which intercalates into the phospholipid bilayer of the cell. They are then rinsed and incubated with antibody, rinsed, and injected. This work was then translated to an *in vivo* application where MSCs were targeted to mucosal vascular addressin cell adhesion molecule (MAdCAM) and VCAM in a dextran sodium sulfate-induced inflammatory bowel model, resulting in a significant improvement in survival [110].

An alternative strategy is the use of bispecific antibodies, whereby an antibody targeting the region of interest is conjugated to an antibody against a ligand expressed on the surface of the cell being delivered. This bispecific antibody approach has been used to target HSCs to damaged vasculature in the heart, using either anti-VCAM-1 conjugated to anti-c-kit [113] or anti-CD45-anti-myosin light chain kinase [112]. This method has also been used in the field of cancer therapy to direct activated T-cells to human epidermal growth factor receptor-2^+^ tumor cells *in vitro* [117]. This cancer treatment was tested *in vivo,* yielding reduced tumor size and a significantly increased rate of survival [118]. *In vitro,* a recent report describes the use of an anti-CD90/anti-myosin light chain kinase to bind MSCs and increase their resistance to shear in a parallel plate assay [119]. 

Of the two antibody-directed cell therapy methods, lipidated protein-G followed by antibody incubation offers the more versatile approach and has been used successfully with MSCs. The same coating technique could, in theory, be applied to any cell type. It is also possible to use multiple antibodies at once [111] and at a higher cell coating density than that achievable with bispecific antibodies because of the limited number of cell surface ligands. Lipidated proteins can also be used to bind other Fc-conjugated ligands; Chen et al. [116] conjugated B7-1 to the Fc portion of human IgG and used it as a co-stimulator. To our knowledge, bispecific antibodies have not been used with an *in vivo *MSC therapy, perhaps because MSCs do not have a cell-specific antibody marker, although any surface-expressed antigen could be utilized. Bispecific antibodies would also have a high cost factor due to difficulties in manufacture and poor stability [120]. 

### 5.3. Genetically-Directed Cell Therapy

Genetically-directed cell therapy is defined as the introduction of genetic material into a cell, either DNA- or RNA-based, to express a ligand on the cell surface that will increase its localization in the target tissue. Genetically overexpressing receptor ligands has great potential, but has the additional hurdles of cell transfection (efficiency, timing, stability, immunogenicity, deleterious effects on cell viability, oncogenic integration sites, and consistent activity), and the regulatory problems associated with delivering both a gene- and cell-therapy. If these issues are overcome, it could be a powerful tool to achieve higher efficacy in cell therapies. Within the MSC targeting field, there have been positive results in cardiac-targeted cells directed using the CXCR4 ligand to induce MSCs to home toward the chemokine SDF1 [121, 122]. Both Cheng et al. [121] and Zhang et al. [122] found increased homing of CXCR4-transduced MSCs to infarcted hearts and improved cardiac output, with Zhang et al. also reporting increased angiogenesis within the infarcted area. Cho et al. found that increasing the expression of CXCR4 also aided in the prevention of bone loss in an ovariectomized mouse model, potentiating the effect of an increased expression of RANK ligand [123]. Another study showed increased homing of CXCR4-transduced C3H10T1/2 cells toward bone marrow along with an increased bone mass in steroid-induced osteoporotic mice, along with complete restoration of bone mass in CXCR4- Cbfa1(RUNX2)-cotransduced cells [124]. Outside of MSC therapy, T-cells have been targeted for anticancer therapy using CD19 [125]. Genetically-modified T-cells have progressed to the clinic with mixed results (for review see [126]).

### 5.4. Selectin-Directed Cell Therapy

Selectin-directed cell targeting was pioneered by Xia et al. [127] who, using *α*-1,3-fucosyltransferase, modified the surface of CD34^+^ cord blood cells and found increased engraftment in NOD/SCID mice. Sackstein et al. [128] applied this technique to modify the cell surface of MSCs, to form HCELL, an E-selectin and L-selectin binding ligand. The use of selectins mimics the natural process of endogenous lymphocyte extravasation. In this process, activated endothelial cells express ligands for selectins, which are endogenously present on lymphocytes, causing them to bind to the endothelium and roll, followed by firm adherence and extravasation [129]. When CD44 on human MSCs was modified to produce the HCELL moiety and injected into immunocompromised mice, the MSCs were observed by intravital microscopy in the calvaria of mice where they extravasated [128]. Sarkar et al. avoided enzymatic modification of the cell surface through two methods: one, biotinylation of cell surface proteins followed by incubation with streptavidin and biotin-conjugated sialyl lewis x (slex), a P-selectin ligand [130, 131], and two, liposome surface modification with biotin-conjugated lipids then incubation with streptavidin and biotin-conjugated slex [132]. It is unclear what benefit Sarkar et al. gained from the more recent liposome method, but it is unlikely that either method would have the same intracellular signaling cascade [133] as that achieved with the enzymatic modification method of Sackstein et al. [128]. Although *in vivo* imaging of selectin-targeted MSCs has been demonstrated by both groups, neither has demonstrated efficacy in a disease model.

### 5.5. Peptide-Directed Cell Therapy

**Figure 3:**
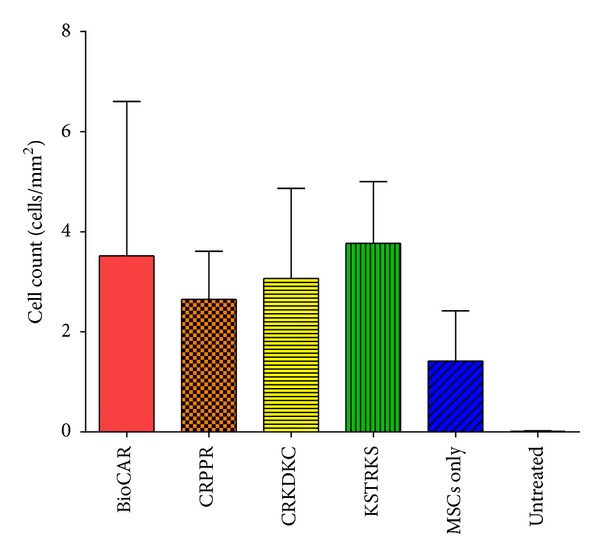
Cell localization in myocardial tissue. Increased cell numbers were found in peptide-targeted (BioCAR, CRPPR, CRKDKC, and KSTRKS) MSCs than in untargeted MSCs (MSCs only). Means of *n* ≥ 3 ± S.D. Total targeted versus untargeted means are 3.1 versus 1.4, *P* < 0.05 one tailed Student's *t*-test.

To our knowledge, we are the only group, to date, that has investigated peptide-directed cell therapy. There has, however, been considerable interest in peptide-targeted drugs for some time, especially in the field of anticancer therapies [134]. Targeting peptides can be derived from endogenous binding proteins, or novel ligands can be identified by combinatorial chemistry libraries or using phage display [134]. Phage display is a powerful method where *in vivo* biopanning experiments can be performed to isolate tissue-specific peptides [135, 136]. This phage display method was utilized by our group with a limited phage “play-off” screen, where several previously identified binding phage peptides were analyzed for their ability to bind to infarcted myocardial tissue [137]. The most successful of these peptides were then synthesized as lipidated derivatives and used to coat MSCs, these were then systemically injected via the left ventricle of MI/reperfusion mice and achieved greater MSC numbers in all peptide-targeted groups than with uncoated MSCs (Figure 3) [137]. However, this increased binding to cardiac tissue was not seen in a permanent ligation model (unpublished results). Peptide-based delivery has several advantages over antibody-, gene-, or selectin-directed techniques: peptide manufacture is scalable, and the products have high purity and are relatively inexpensive to produce. Ligands can be highly specific for tissues or cells of interest and multiple ligands can be attached to the cell surface or drug delivery vehicle. However, in contrast to antibody-based therapies, no peptide-targeted therapeutic has yet made it to market [138].

## 6. Summary

MSCs are currently being used in clinical trials for the treatment of a range of diseases, with varying degrees of efficacy. At this point, there is no study that we know of that has demonstrated efficient long-term engraftment of MSCs, whether locally or systemically injected, and it is hypothesized that the therapeutic efficacy of delivered MSCs will increase dramatically if MSCs can be made to engraft more efficiently by directing them to the site(s) of the lesion(s) to be treated. The loss of locally injected cells can be attributed to wash out, cell death, and, perhaps, rejection via the innate immune system or, if the MSCs begin to differentiate, they may become targets of the adaptive immune system. For systemic delivery, IV delivery is the most common mode of introduction. At the same time, IV infusion has been shown to result in the entrapment of MSCs in the lung, if only temporarily, which negatively impacts engraftment, and several studies have shown that IA injection is a more effective means of delivering MSCs. Methods to more effectively target MSCs to tissues and organs are being developed, including the coating of cells with antibodies or peptides, modifying native cell surface molecules into endothelium-binding molecules, genetic modification, or biotinylating cell surface molecules and then coating the cells with streptavidin and biotinylated antibodies. Future improvements in the targeting methodologies combined with IA injection have the potential to increase engraftment and improve therapeutic efficacy while, at the same time, reducing the ECD needed.
